# Effects and mechanisms of different exercise modalities on inflammation in older adults, particularly with sarcopenia: a narrative review

**DOI:** 10.3389/fmed.2026.1819905

**Published:** 2026-06-08

**Authors:** Yanxia Shang, Yanfeng Shang

**Affiliations:** 1 School of Athletic Performance, Shanghai University of Sport, Shanghai, China; 2 Department of Physical Education and Health, Wuxi Vocational Institute of Commerce, Wuxi, Jiangsu, China

**Keywords:** aging, exercise modalities, inflammation, molecular mechanisms, resistance training, sarcopenia

## Abstract

Sarcopenia is a prevalent age-related muscle disorder characterized by progressive loss of muscle mass and function, and chronic low-grade inflammation (“inflammaging”) is recognized as a key contributor to its pathogenesis. Exercise has been proposed as an effective non-pharmacological strategy to counteract inflammation and improve muscle health; however, the anti-inflammatory effects and underlying mechanisms of different exercise modalities remain incompletely understood. This review aims to summarize and compare the effects of different exercise modalities on inflammatory responses in older adults with sarcopenia and to elucidate the potential molecular and cellular mechanisms involved. A narrative review was conducted by searching PubMed, Web of Science, and Scopus for relevant studies published in the past decade. Clinical trials and experimental studies investigating aerobic exercise, resistance training, high-intensity interval training, and combined training interventions in older adults with sarcopenia were included. Outcomes of interest focused on systemic and muscle-derived inflammatory markers, including tumor necrosis factor-α, interleukin-6, C-reactive protein, and anti-inflammatory cytokines. Aerobic exercise predominantly reduces systemic inflammatory markers and improves metabolic regulation, whereas resistance training mainly attenuates muscle-derived inflammatory signaling and promotes anabolic responses. High-intensity interval training and combined training programs appear to exert complementary effects on both systemic and local inflammation. At the molecular level, exercise-induced anti-inflammatory effects are associated with suppression of pro-inflammatory pathways such as nuclear factor-κB and toll-like receptor 4, and activation of regulatory pathways including AMP-activated protein kinase/sirtuin 1 and nuclear factor erythroid 2–related factor 2. In addition, exercise modulates myokine secretion and immune cell phenotypes, contributing to an improved inflammatory microenvironment in skeletal muscle. Different exercise modalities exert distinct but overlapping anti-inflammatory effects in older adults with sarcopenia. Understanding the specific inflammatory targets and mechanisms of various exercise interventions may facilitate the development of individualized and optimized exercise prescriptions for the prevention and management of sarcopenia.

## 1 Introduction

With the rapid acceleration of global population aging, sarcopenia has emerged as a major public health concern. Defined as an age-related geriatric syndrome characterized by progressive loss of skeletal muscle mass, strength, and physical performance, sarcopenia is now widely recognized according to the criteria established by the European Working Group on Sarcopenia in Older People (EWGSOP) and the Asian Working Group for Sarcopenia (AWGS) (1, 2). These impairments substantially increase the risk of falls, fractures, disability, and mortality, thereby significantly compromising quality of life and independence among older adults (1, 2).

Chronic low-grade inflammation plays a pivotal role in the onset and progression of sarcopenia. Aging is accompanied by a persistent inflammatory state characterized by elevated circulating levels of inflammatory mediators, including IL-6, tumor necrosis factor-α (TNF-α), and C-reactive protein (CRP). This phenomenon, often referred to as “inflammaging,” contributes to sarcopenia by disrupting protein synthesis, promoting muscle catabolism, and impairing muscle regenerative capacity (3). Accordingly, modulation of inflammatory status has been considered an important therapeutic target for sarcopenia.

Substantial evidence demonstrates that appropriately prescribed exercise can enhance muscle mass and strength and may also improve the course of sarcopenia by regulating immune and inflammatory responses (4, 5). In particular, resistance training, aerobic exercise, and their combined forms have attracted increasing attention in recent years. However, the specific effects of different exercise modalities on sarcopenia-related inflammatory markers and their underlying mechanisms remain controversial and incompletely understood.

Several studies have reported that exercise can significantly reduce levels of pro-inflammatory cytokines, such as IL-6 and TNF-α (6, 7), whereas others have failed to observe significant changes (8), suggesting that the anti-inflammatory effects of exercise may be influenced by multiple factors, including exercise intensity, frequency, and individual nutritional status. Therefore, a systematic synthesis of existing evidence is warranted.

The present review aims to comprehensively summarize and analyze recent findings on the effects of different exercise modalities on inflammatory markers in older adults with sarcopenia and to explore the potential molecular mechanisms involved. From the perspective of inflammatory regulation, this review seeks to provide a theoretical framework and practical implications for optimizing exercise-based intervention strategies for sarcopenia.

## 2 Methods

### 2.1 Search strategy

This review was conducted following a structured approach to enhance transparency. Electronic databases including PubMed, Web of Science, and Scopus were systematically searched for studies published between January 2010 and January 2026.

The search strategy combined Medical Subject Headings (MeSH) and free-text terms: (“sarcopenia” OR “muscle loss” OR “age-related muscle decline”) AND (“exercise” OR “physical activity” OR “resistance training” OR “aerobic training” OR “high-intensity interval training”) AND (“inflammation” OR “cytokines” OR “TNF-α” OR “IL-6” OR “CRP”).

### 2.2 Inclusion and exclusion criteria

Studies were included if they:

(1) involved older adults (≥60 years) or individuals with sarcopenia;
(2) examined exercise interventions;
(3) reported inflammatory outcomes.

Exclusion criteria included:

(1) Duplicate publications;
(2) Studies with inaccessible full texts or incomplete data reporting;
(3) studies lacking relevant inflammatory outcomes.

A total of 60 studies were included after screening.

### 2.3 Study selection

All identified records were imported into reference management software, and duplicates were removed. Titles and abstracts were screened for relevance, followed by full-text assessment based on the predefined inclusion and exclusion criteria. The study selection process was conducted independently by two reviewers, and disagreements were resolved through discussion.

**Figure 1:**
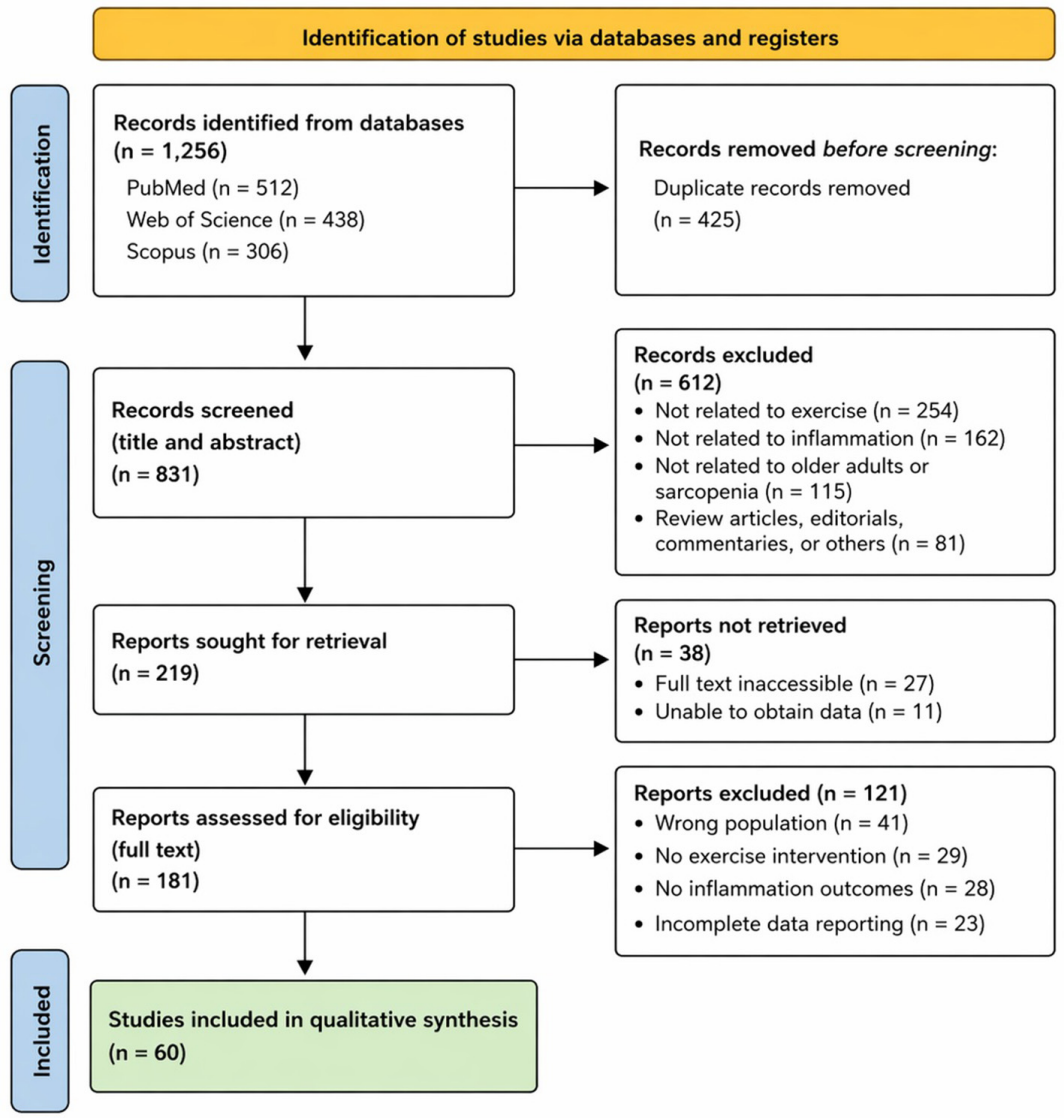
Flow diagram of literature screening and selection according to the PRISMA statement. This figure follows the PRISMA 2020 guidelines. *n*, number of records or studies.

Although this study is a narrative review, the selection process followed a structured approach similar to the Preferred Reporting Items for Systematic Reviews and Meta-Analyses (PRISMA) guidelines. A flow diagram illustrating the study selection process is provided in Figure 1.

### 2.4 Data extraction and synthesis

Key information from the included studies was extracted, including author, year of publication, study design, participant characteristics, type of exercise intervention, duration and intensity of exercise, and main inflammatory outcomes.

Due to the heterogeneity in study designs, populations, and outcome measures, a qualitative synthesis was conducted. The findings were organized into thematic categories based on different exercise modalities and their associated anti-inflammatory mechanisms.

## 3 Results

### 3.1 Overview of included studies

A total of 60 studies were included in this review after the screening process, including randomized controlled trials, observational studies and reviews. The exercise interventions varied considerably across studies in terms of type, intensity, frequency, and duration. The primary inflammatory outcomes included circulating cytokines such as interleukin-6 (IL-6), tumor necrosis factor-alpha (TNF-α), and C-reactive protein (CRP).

### 3.2 Effects of aerobic exercise on inflammation

Aerobic exercise interventions, including walking, cycling, and treadmill training, were commonly investigated. Most studies reported that moderate-intensity aerobic exercise was associated with reductions in pro-inflammatory markers such as CRP and TNF-α (9–12).

Some studies also observed transient increases in IL-6 immediately following acute exercise sessions, while long-term aerobic training tended to reduce baseline IL-6 levels (9, 12). The duration of interventions ranged from several weeks to several months, with more consistent anti-inflammatory effects observed in longer-duration programs (10).

### 3.3 Effects of resistance training on inflammation

Resistance training was another frequently studied modality, particularly in populations with sarcopenia. The interventions typically involved progressive resistance exercises targeting major muscle groups (13, 14).

Most studies indicated that resistance training reduced systemic inflammation, as evidenced by decreased levels of CRP and TNF-α (15–17). Some studies also reported improvements in muscle mass and strength alongside reductions in inflammatory markers (18, 19). However, the magnitude of changes varied depending on training intensity and participant characteristics (20, 21).

### 3.4 Effects of combined exercise interventions

Combined exercise programs incorporating both aerobic and resistance training were also examined. These interventions often demonstrated more pronounced or consistent anti-inflammatory effects compared to single-modality programs (12, 22).

Reductions in CRP and TNF-α were commonly reported (23, 24), and some studies suggested synergistic benefits of combining different exercise modalities (25, 26). Intervention durations and protocols varied widely, but multi-component programs were generally associated with broader physiological improvements (23, 25).

The effects of different exercise modalities on inflammatory markers in older adults with or without sarcopenia are summarized below (Table1).

**Table 1:** Heterogeneity of exercise interventions and inflammatory outcomes in older adults with or without sarcopenia.

References	Population	Modality	Intensity	Duration	Frequency	Markers	Main findings
Forti et al. (18)	Community-dwelling older adults	Resistance	Moderate	12 weeks	2–3/w	IL-6	↓IL-6
Nunes et al. (20)	Postmenopausal women	Resistance	Moderate	12–16 weeks	3/w	CRP, TNF-α	↓CRP ↓TNF-α
Chen et al. (64)	Sarcopenic elderly women	Resistance	Moderate	8 weeks	3/w	IL-6, TNF-α	↓IL-6 ↓TNF-α
Perreault et al. (65)	Sarcopenic men	Resistance	Moderate	16 weeks	3/w	CRP, IL-6	No significant change
de Sá Souza et al. (66)	Sarcopenic older adults	Resistance	Moderate	12 weeks	3/w	IL-10, TNF-α	↑IL-10 ↓TNF-α
Dong et al. (16)	Hemodialysis patients with sarcopenia	Resistance	Moderate	12 weeks	3/w	CRP, IL-6	↓CRP ↓IL-6
Gadelha et al. (17)	CKD older adults	Resistance	Moderate	12 weeks	2–3/w	IL-6, TNF-α	↓IL-6 ↓TNF-α
Heo et al. (21)	Older adults	Resistance	Low vs. High	12 weeks	3/w	IL-6, TNF-α	Intensity-dependent response
Yuenyongchaiwat et al. (25)	Sarcopenic older adults	Combined (walking + RT)	Moderate	12 weeks	3/w	IL-6, TNF-α	↓IL-6 ↓TNF-α
Park et al. (24)	Osteoarthritis + sarcopenia	Combined	Moderate	12 weeks	3/w	IL-6, IL-10	↓IL-6 ↑IL-10
Kim et al. (67)	Older adults	Combined	Moderate	6 weeks	3/w	Chemerin	↓inflammatory marker
Wanderley et al. (68)	Older adults	Aerobic vs Resistance	Moderate	12 weeks	3/w	CRP, IL-6	Aerobic > RT for CRP reduction
Irwin et al. (69)	Older adults	Tai Chi	Low-Moderate	16 weeks	2–3/w	CRP	↓CRP
Alghadir et al. (11)	Older adults	Aerobic	Moderate	12 weeks	3/w	Oxidative stress markers	↓inflammation-related markers
Zhuang et al. (70)	Sarcopenic older adults	WBV vs. RT	Moderate	12 weeks	3/w	IL-6	↓IL-6 (both groups)

## 4 Discussion

Overall, the present review demonstrates that different exercise modalities exert heterogeneous effects on inflammatory markers in older adults with sarcopenia. Resistance training and combined exercise appear to provide relatively more consistent anti-inflammatory benefits, particularly in reducing CRP and improving cytokine balance, whereas the effects of aerobic exercise remain variable. Importantly, the anti-inflammatory effects of exercise are influenced by multiple factors, including training intensity, duration, baseline inflammatory status, and nutritional conditions.

Chronic low-grade inflammation is widely recognized as a central pathological mechanism underlying age-related sarcopenia. Persistent elevations in pro-inflammatory cytokines, including IL-6, TNF-α, and CRP, contribute to muscle protein degradation, impaired protein synthesis, and dysfunction of satellite cells, ultimately leading to progressive loss of muscle mass and function (27–29). In particular, sarcopenia frequently coexists with obesity, where increased infiltration of pro-inflammatory immune cells in adipose tissue further amplifies systemic inflammation and accelerates muscle catabolism (30, 31).

At the molecular level, inflammatory signaling pathways such as NF-κB, JAK/STAT, and FoxO play critical roles in mediating muscle atrophy. TNF-α activates NF-κB signaling, inducing the expression of muscle atrophy-related genes such as MuRF1 and Atrogin-1, while IL-6 promotes proteolysis and suppresses protein synthesis *via* JAK/STAT signaling (32, 33). In addition, inflammation-induced inhibition of Akt signaling further enhances FoxO-mediated protein degradation and antagonizes anabolic IGF-1 signaling (34). Emerging evidence also suggests that inflammatory pathways may induce pyroptosis through activation of the NLRP3 inflammasome, thereby exacerbating muscle injury (35, 36).

Importantly, inflammatory mediators exhibit context-dependent effects. Although chronically elevated IL-6 is associated with muscle loss, exercise-induced transient increases in IL-6 may exert anti-inflammatory effects by stimulating IL-10 and IL-1 receptor antagonist production, thereby contributing to immune regulation (37, 38). This dual role of IL-6 highlights the importance of distinguishing between chronic inflammation and acute exercise-induced responses when interpreting findings. Anti-inflammatory cytokines such as IL-10 and TGF-β further facilitate muscle repair by promoting immune balance and regulating satellite cell activity (39–42). However, excessive activation of TGF-β signaling may impair muscle regeneration and promote fibrosis, indicating its dual regulatory role in skeletal muscle remodeling (42–44).

**Figure 2:**
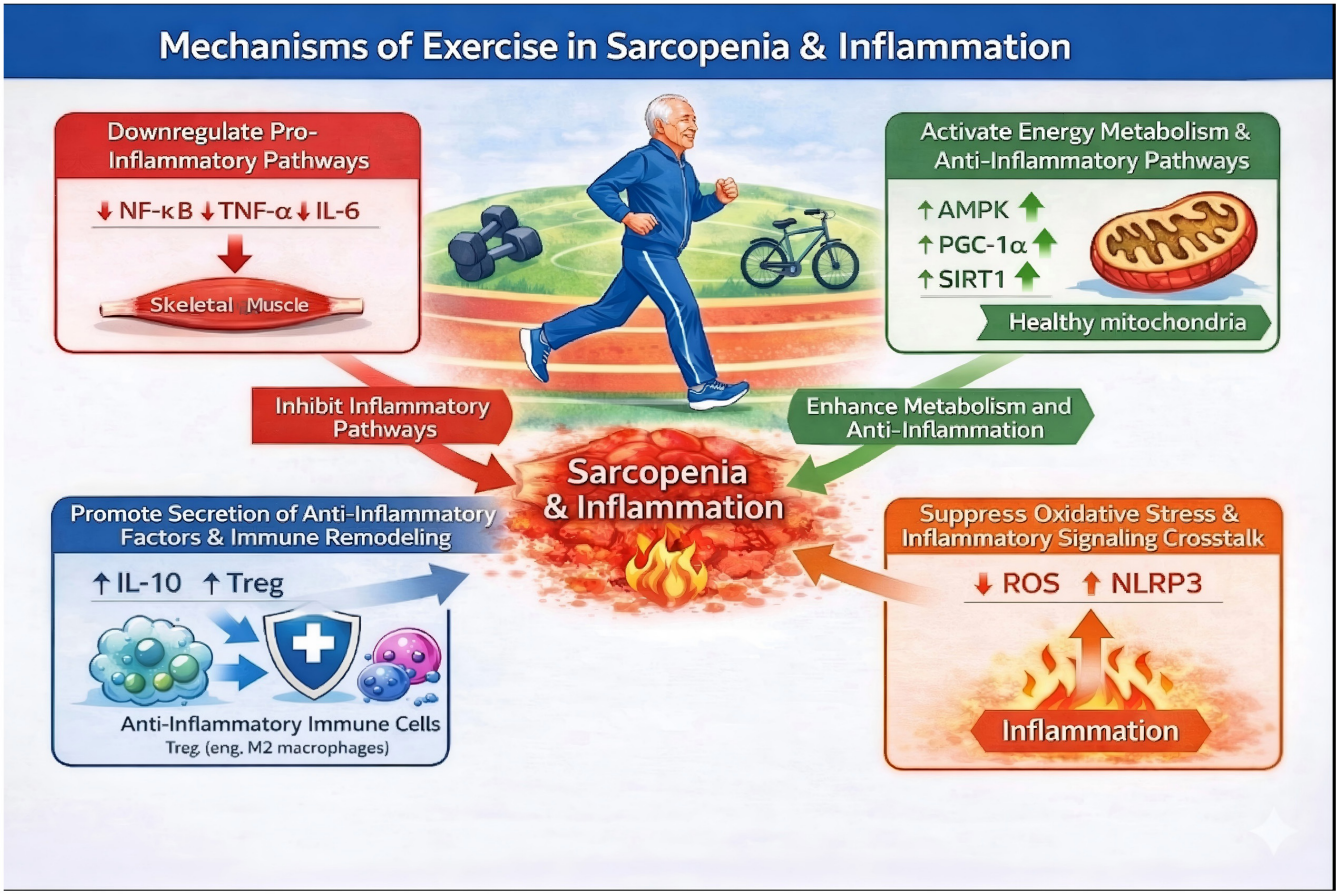
Exercise-induced mechanisms for reducing inflammation in sarcopenia.

Building upon these mechanisms, exercise interventions may modulate the inflammatory microenvironment through multiple interconnected pathways (Figure 2). In particular, exercise can downregulate classical pro-inflammatory signaling cascades, including TLR4/NF-κB and JAK/STAT pathways, thereby reducing cytokine production and attenuating muscle proteolysis (45–51). In parallel, exercise activates key metabolic regulators such as the AMPK–SIRT1–PGC-1α axis, which enhances mitochondrial function, improves energy metabolism, and suppresses NF-κB-mediated inflammatory signaling (52–56).

In addition, exercise promotes anti-inflammatory cytokine secretion and immune remodeling. Acute exercise-induced IL-6 acts as a myokine to stimulate IL-10 and IL-1 receptor antagonist production, forming an anti-inflammatory cascade (57). Exercise also induces shifts in immune cell phenotypes, including macrophage polarization toward an anti-inflammatory M2 phenotype, which contributes to improved muscle regeneration and reduced inflammation (57, 58, 58–60). Furthermore, exercise modulates oxidative stress–inflammation crosstalk by enhancing antioxidant defense systems and reducing reactive oxygen species (ROS)-mediated activation of inflammatory pathways such as NF-κB (58, 61–63). Collectively, these adaptations suggest that exercise functions as a key regulator of the muscle–immune–metabolic axis in sarcopenia.

### 4.1 Resistance training

Resistance training is widely regarded as the cornerstone intervention for sarcopenia due to its well-established effects on muscle hypertrophy and strength. Increasing evidence suggests that resistance training also exerts anti-inflammatory effects, particularly when combined with nutritional interventions. Several randomized controlled trials have demonstrated reductions in IL-6 and TNF-α following resistance training combined with protein supplementation or vitamin D, whereas resistance training alone often produces more modest or non-significant changes (13–71). These effects may be mediated by suppression of NF-κB signaling and improved muscle–immune interactions, and are further enhanced by optimized nutritional status (72–74).

In addition to reducing pro-inflammatory cytokines, resistance training may promote anti-inflammatory pathways. Increases in IL-10 and IL-1 receptor antagonist have been observed even in the absence of significant reductions in IL-6 or TNF-α, suggesting that exercise may exert its benefits by shifting cytokine balance rather than solely suppressing pro-inflammatory markers (66). Similar effects have been reported in clinical populations, including patients with chronic kidney disease, where resistance exercise reduced CRP levels and improved inflammatory profiles (16, 17).

Nevertheless, findings across studies remain inconsistent. While some trials report significant reductions in IL-6 or CRP (18–20), others show no significant changes (65, 75). Meta-analyses indicate relatively consistent reductions in CRP but heterogeneous effects on IL-6 and TNF-α (76, 77). These discrepancies may be explained by differences in training intensity, volume, and duration. For example, moderate-intensity resistance training appears more effective in reducing IL-6, whereas higher intensity may preferentially influence TNF-α (21), suggesting cytokine-specific sensitivity to exercise stimuli.

### 4.2 Aerobic exercise

Aerobic exercise is widely used to improve cardiovascular and metabolic health and has demonstrated potential anti-inflammatory effects. Moderate-intensity aerobic exercise has been associated with reductions in CRP, TNF-α, and IL-6 in older adults (9–12). Mechanistically, these effects may be related to activation of AMPK signaling, improved mitochondrial function, and reduced oxidative stress.

However, much of this evidence is derived from non-sarcopenic populations, limiting its direct applicability to individuals with sarcopenia. In older adults, aerobic exercise appears to consistently reduce CRP levels, whereas its effects on IL-6 are less consistent (10–12). Some evidence suggests that aerobic exercise may attenuate age-related increases in inflammatory markers rather than produce substantial absolute reductions (68), indicating a role in maintaining inflammatory homeostasis.

The effectiveness of aerobic exercise depends strongly on intervention characteristics. Insufficient intensity or duration may fail to induce significant changes, whereas longer-term interventions are more likely to reduce chronic low-grade inflammation. Additionally, baseline inflammatory status may influence responsiveness.

### 4.3 Combined exercise

Combined exercise, integrating resistance and aerobic components, is frequently recommended in sarcopenia management because it simultaneously targets muscle strength and cardiometabolic function. Emerging evidence suggests that combined exercise may exert broader anti-inflammatory effects than single-modality interventions (22).

Meta-analytic findings indicate that combined exercise significantly reduces CRP and IL-6, whereas aerobic exercise primarily affects TNF-α and CRP, and resistance training mainly reduces CRP (12). These effects may reflect the synergistic activation of multiple pathways, including improved muscle protein metabolism and enhanced mitochondrial function. Randomized controlled trials have shown that combined exercise can substantially reduce CRP levels and improve overall inflammatory status (23, 25). In clinical populations, combined exercise has been associated with increased IL-10 and decreased TNF-α, indicating coordinated regulation of pro- and anti-inflammatory pathways (24, 26).

However, not all studies report positive findings. Several trials have found no significant changes in IL-6 or CRP following combined interventions (27–63, 67–80). These inconsistencies may be attributed to insufficient intervention duration, low training frequency, or relatively low baseline inflammation. Additionally, improvements in cytokine balance, such as the TNF-α/IL-10 ratio, may be more informative than isolated changes in individual markers (81).

### 4.4 Other exercise modalities

Emerging exercise modalities, including Tai Chi, high-intensity interval training (HIIT), yoga, and whole-body vibration (WBV), have shown potential benefits in regulating inflammatory responses in older adults. Tai Chi has been associated with reductions in CRP and IL-6 and improvements in functional performance, possibly through modulation of autonomic function and stress-related pathways (69).

HIIT may activate AMPK/SIRT1 signaling and induce rapid metabolic adaptations, thereby contributing to anti-inflammatory effects (82). Whole-body vibration has demonstrated comparable reductions in CRP, IL-6, and TNF-α to traditional resistance training, suggesting its feasibility for individuals with limited exercise tolerance (70, 83).

Mind–body interventions such as yoga may reduce cortisol levels and modulate NF-κB signaling, thereby improving immune regulation (84, 85). Although evidence remains limited for some modalities, these findings highlight the diversity of exercise strategies capable of modulating inflammatory pathways.

### 4.5 Sources of heterogeneity

A major challenge in interpreting the effects of exercise on inflammatory markers in sarcopenia is the substantial heterogeneity across studies. Differences in exercise modality, intensity, duration, and frequency, as well as variability in participant characteristics, contribute to inconsistent findings.

From a mechanistic perspective, aerobic exercise primarily enhances mitochondrial function and activates anti-inflammatory pathways such as AMPK and PGC-1α, whereas resistance training promotes muscle protein synthesis and improves muscle quality. High-intensity exercise may induce transient increases in pro-inflammatory cytokines due to acute physiological stress, followed by adaptive anti-inflammatory responses.

Participant-related factors also play a critical role. Nutritional status, obesity, and comorbidities such as diabetes and chronic kidney disease are closely associated with chronic inflammation and may influence responsiveness to exercise interventions (16, 17, 41, 73, 81, 86, 87). Baseline physical activity levels further modulate inflammatory responses, with more active individuals typically exhibiting lower baseline inflammation and greater adaptive capacity (23). These factors collectively contribute to the heterogeneity observed across studies and underscore the necessity of individualized and precision-based exercise prescriptions.

### 4.6 Clinical implications

From a clinical perspective, these findings support the use of individualized exercise prescriptions in the management of sarcopenia. Resistance training should be considered the cornerstone intervention due to its combined anabolic and anti-inflammatory effects, while combined exercise may provide additional benefits in regulating systemic inflammation and improving overall functional capacity.

For example, moderate-intensity resistance training performed at least 2–3 times per week may represent a practical and effective strategy for reducing chronic inflammation in older adults. Furthermore, integrating exercise with nutritional strategies, such as protein supplementation, vitamin D, or omega-3 fatty acids, may enhance anti-inflammatory effects and optimize intervention outcomes. These approaches align with current concepts of integrated “exercise–nutrition” interventions.

### 4.7 Strengths and limitations

This review has several strengths. It provides a comprehensive synthesis of the effects of different exercise modalities on inflammation in sarcopenic older adults and integrates findings from both clinical and experimental studies.

However, several limitations should be acknowledged. First, inflammatory markers are often reported as secondary outcomes, and the selection of biomarkers varies across studies, limiting comparability. Second, heterogeneity in exercise protocols and participant characteristics complicates interpretation of results. Third, most studies are of relatively short duration, and long-term effects of exercise on inflammation in sarcopenia remain unclear.

## 5 Conclusion

In summary, chronic low-grade inflammation represents a central mechanism underlying the development and progression of age-related sarcopenia, contributing to muscle protein degradation, impaired regeneration, and functional decline. The present review demonstrates that exercise interventions play a pivotal role in modulating inflammatory responses through multiple interconnected pathways.

Different exercise modalities exhibit heterogeneous effects on inflammatory markers. Resistance training and combined exercise appear to provide relatively more consistent anti-inflammatory benefits, particularly in improving cytokine balance and reducing systemic inflammatory burden, whereas the effects of aerobic exercise remain variable and may depend on intervention characteristics and baseline conditions.

At the mechanistic level, exercise regulates inflammation through suppression of classical pro-inflammatory signaling pathways (e.g., NF-κB and JAK/STAT), activation of metabolic regulators (e.g., AMPK–SIRT1–PGC-1α axis), promotion of anti-inflammatory cytokine responses, and modulation of oxidative stress–inflammation crosstalk. These adaptations collectively highlight the role of exercise as a key modulator of the muscle–immune–metabolic axis in sarcopenia.

From a clinical perspective, these findings support the implementation of individualized exercise prescriptions, with resistance training as a cornerstone strategy and combined exercise as a complementary approach. Integration of exercise with nutritional interventions may further enhance anti-inflammatory and functional outcomes.

However, the heterogeneity of study designs, variability in inflammatory biomarkers, and limited availability of long-term and mechanistic human studies remain important challenges. Future research should focus on well-designed randomized controlled trials, standardized assessment of inflammatory outcomes, and deeper exploration of dose–response relationships to optimize exercise-based strategies for sarcopenia management.
